# Using dual-task gait to recognize Alzheimer’s disease and mild cognitive impairment: a cross-sectional study

**DOI:** 10.3389/fnhum.2023.1284805

**Published:** 2023-12-19

**Authors:** Zhaoying Li, Jingyi Zhu, Junyan Liu, Min Shi, Pan Liu, Junjie Guo, Zhenzhu Hu, Shanyu Liu, Dongdong Yang

**Affiliations:** Department of Neurology, Hospital of Chengdu University of Traditional Chinese Medicine, Chengdu, Sichuan, China

**Keywords:** arm motion, asymmetry, gait, mild cognitive impairment, Alzheimer’s disease

## Abstract

**Background:**

Gait is a potential diagnostic tool for detecting mild cognitive impairment (MCI) and Alzheimer’s disease (AD). Nevertheless, little attention has been paid to arm movements during walking, and there is currently no consensus on gait asymmetry. Therefore, in this study, we aimed to determine whether arm motion and gait asymmetry could be utilized for identifying MCI and AD.

**Methods:**

In total, 102 middle-aged and elderly individuals were included in the final analysis and were assigned to the following three groups: AD (*n* = 27), MCI (*n* = 35), and a normal control group (*n* = 40). Gait and cognitive assessments were conducted for all participants. Gait detection included a single-task gait with free-speed walking and a dual-task gait with adding a cognitive task of successive minus seven to walking. Original gait parameters were collected using a wearable device featuring the MATRIX system 2.0. Gait parameters were shortened to several main gait domains through factor analysis using principal component extraction with varimax rotation. Subsequently, the extracted gait domains were used to differentiate the three groups, and the area under the receiver operating characteristic curve was calculated.

**Results:**

Factor analysis of single-task gait identified five independent gait domains: rhythm symmetry, rhythm, pace asymmetry, arm motion, and variability. Factor analysis of the dual-task gait identified four gait domains: rhythm, variability, symmetry, and arm motion. During single-task walking, pace asymmetry was negatively correlated with MoCA scores and could distinguish between the AD group and the other two groups. Arm motion was not associated with MoCA scores, and did not exhibit adequate discrimination in either task.

**Conclusion:**

Currently, there is no reliable evidence suggesting that arm motion can be used to recognize AD or MCI. Gait asymmetry can serve as a potential gait marker for the auxiliary diagnosis of AD but not for MCI.

## 1 Introduction

Alzheimer’s disease (AD) is one of the main causes of disability and death in the elderly (Alzheimer’s Association, 2023). Mild cognitive impairment (MCI) is a transitional cognitive state that occurs between normal aging and dementia, and the conversion rate of MCI patients to AD is approximately 10–15% per year (Ghoraani et al., 2021; Alzheimer’s Association, 2023). Owing to the lack of effective treatment, the early detection of AD and MCI is essential for slowing progression. Compared to cerebrospinal fluid markers and imaging examinations, gait detection is easier to operate, cheaper, and more portable and intuitive, all of which make gait markers highly applicable. Gait abnormalities are common in patients with Alzheimer’s disease (AD), and can also predict a higher dementia risk in individuals with mild cognitive impairment (MCI) (Montero-Odasso et al., 2017, 2020). Shared cortical networks may partially explain the correlation between cognitive impairment and gait impairment (Takakusaki, 2023). Spatial navigation and visuospatial abilities, which are potentially impaired in individuals with AD and MCI, may also affected postural control during gait (Cohen and Verghese, 2019; Plácido et al., 2022; Chepisheva, 2023). Several gait markers have been identified in individuals with AD and MCI, including high variability, slow walking speed, and decreased stride length (Yang et al., 2020; Boripuntakul et al., 2022; Lindh-Rengifo et al., 2022). However, arm movements during walking are rarely observed.

Arm swing during walking contributes to gait coordination and stability (Meyns et al., 2013). Gait instability is prevalent in individuals with AD and MCI, increasing the risk of falls (Sheridan and Hausdorff, 2007; Auvinet et al., 2017). In healthy adults, arm swing amplitude is reduced when performing an executing cognitive task during walking, and an increase in the asymmetry of arm movement is also observed (Mirelman et al., 2015; Killeen et al., 2017). A recent study showed that upper-body movements are highly weighted in the gait diagnostic models used for AD (You et al., 2021). Therefore, we focused on the arm motion in individuals with AD and MCI, and examined whether it could serve as a potential biomarker for AD and MCI.

Furthermore, gait asymmetry in patients with AD and MCI has not been fully explored, as it has previously been suggested responding to changes in unilateral pathology (Mc Ardle et al., 2020; Di Biase et al., 2022). In a recent study, a significant increase in step length asymmetry was detected in individuals with MCI and AD compared to healthy individuals (Ghoraani et al., 2021). However, whether gait asymmetry can identify AD or MCI as an distinct gait domain remains unknown.

Therefore, in this cross-sectional study, we sought to investigate the potential of arm movement and gait asymmetry in identifying AD and MCI. We hypothesized that changes in arm swing and gait asymmetry could be detected in patients with AD and MCI and could be used to distinguish between different cognitive states.

## 2 Materials and methods

### 2.1 Study design and recruitment

This cross-sectional controlled study employed a convenience sampling method. Patients with AD or MCI from the Dementia Clinic of the Hospital of Chengdu University of Traditional Chinese Medicine were invited to participate in this study from June to December 2022. Cognitively healthy individuals were recruited from a health screening center. We calculated the minimum sample size based on the following formula: *n* = ${\left(\frac{Z_{1-\alpha }/2^{*\sigma }}{\delta }\right)}^{2}$, and referred to the results of a previous study (Muir et al., 2012). The minimum sample sizes for the AD and MCI groups were 18 and 17, respectively. All participants volunteered to participate in this study and were informed of its objectives, content, risks, and benefits before data collection. All participants underwent cognitive assessments and gait detection performed by three experienced neurologists. This study (2022KL-042) was approved by the Ethics Committee of the Hospital of Chengdu University of Traditional Chinese Medicine.

### 2.2 Participants

**FIGURE 1:**
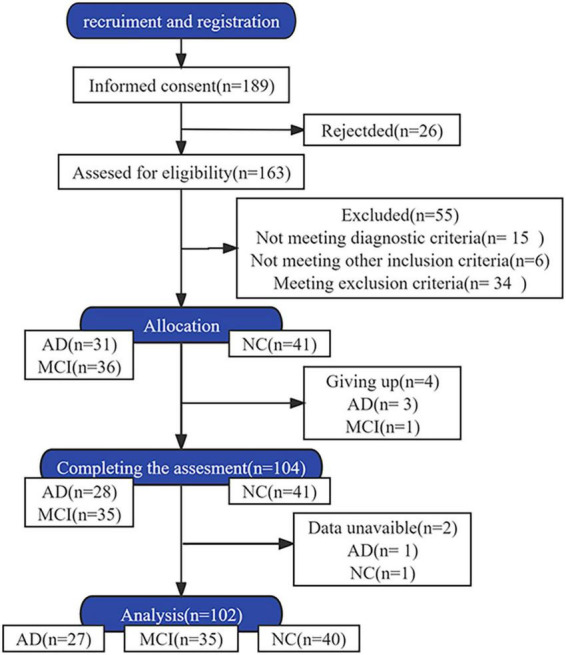
Study flow diagram.

Patients with AD and MCI, and healthy individuals aged 45–85 years were included. All participants were able to walk independently without instrumental aids. AD and MCI were diagnosed by Min Shi and Dongdong Yang, respectively. The diagnostic criteria for AD conformed to the clinical diagnostic criteria for “probable AD dementia” established by the National Institute of Aging-Alzheimer’s Association (McKhann et al., 2011). The clinical dementia rating scores for participants with AD ranged from 1 to 2. The diagnostic criteria for MCI lined with the expert consensus of the International Working Group on MCI (Winblad et al., 2004). The exclusion criteria for all participants included: speech impairment, other neurological disorders, other types of dementia, musculoskeletal disorders, history of knee or hip replacement affecting gait performance, consumption of psychotropic drugs affecting exercise capacity, psychiatric disorders, and severe diseases of other vital organs. The participants were allowed to discontinue the study at any time during the experiment. Participants with incomplete data were excluded from the analysis. The participant screening process is illustrated in Figure 1.

### 2.3 Clinical characteristic collection

The participants’ basic characteristics included age, sex, weight, height, body mass index, and years of education. The Mini-Mental State Examination (MMSE) and Montreal Cognitive Assessment (MoCA) were used to evaluate cognition. The MMSE is the most widely used standardized cognitive assessment test in clinical practice (Ciesielska et al., 2016; Gallegos et al., 2022). The MoCA covers broader cognitive domains than the MMSE and is designed to screen for MCI with high sensitivity and specificity (Nasreddine et al., 2005).

### 2.4 Gait assessment

Three neurologists performed gait assessments to collect the gait parameters using a wearable device (Gyenno Science Co., Ltd., MATRIX system2.0). The device included ten inertial measurement unit sensors and a host computer loaded with a quantitative gait assessment system. Sensors were worn on the tester’s chest, waist, hands, thighs, calves, and feet. The raw kinematic signals of the participants were captured using sensors and transmitted to the host computer via a Bluetooth link for further analysis.

The participants were instructed to complete the Timed Up and Go (TUG) test for single-task (ST) and dual-task (DT). Verbal guidance was provided during the testing. Each participant practiced before the formal trial. The TUG test consists of three consecutive movements of getting up, turning around, and walking and provides a good assessment of walking ability and balance (Podsiadlo and Richardson, 1991). TUG of ST: After hearing the “start” command, the participant walked freely along a straight line to the “3 meter” marker at a comfortable speed, turned around, and walked back to the beginning. There was 1 m of free walking space after the “3 meter” marker, allowing acceleration, deceleration and turning around. TUG of DT: The participants were asked to perform consecutive minus seven calculations starting from 100 while performing the TUG test. Cognitive and motor tasks were not prioritized.

### 2.5 Gait parameters

The wearable device directly generates a series of objective gait parameters. From these, we selected 20 parameters (see Supplementary Table 1) to analyze the mean value, asymmetry, variability and arm swinging while walking. The parameters were selected with reference to a previous gait model in older adults (Lord et al., 2013), and other recent relevant studies (Byun et al., 2018; Ghoraani et al., 2021; Lindh-Rengifo et al., 2022; Zheng et al., 2022).

### 2.6 Data analysis

SPSS version 26.0 (IBM Corp., Armonk, NY, USA) was used for all analyses. The participant characteristics were descriptively analyzed in the AD, MCI, and NC groups. Normally distributed continuous variables were explored using an one-way analysis of variance. Abnormally distributed variables were analyzed using the Kruskal–Wallis tests. Gait parameters were descriptively analyzed using analysis of covariance. Age and years of education were included as covariates. Natural log transformation was used to improve the normality of gait parameters. Bonferroni’s correction states were used for *post hoc* comparisons. *P* < 0.05 was considered a significant difference between the groups.

Factor analysis (FA) was used to reduce the 20 gait parameters to several main gait domains using principal component (PC) analysis with varimax rotation. PCs with eigenvalues ≥1 were selected. Gait parameters with positive or negative loads ≥0.5 were considered relevant component contributors (Costello and Osborne, 2005). Subsequently, linear regression models were constructed to explore the correlation between the gait domains and cognitive performance. Finally, we examined the diagnostic effect of the gait domains between different cognitive states using receiver operating characteristic (ROC) curves, and the areas under the curve (AUC) were calculated. For gait domains with potential diagnostic value, ROC curves adjusted for age and education were also constructed (Janes et al., 2009). The best cut-off value was determined based on the largest Youden index. Sensitivity, specificity, and 95% confidence intervals (CI) were reported.

## 3 Results

### 3.1 Participant characteristics

In total, 102 participants were enrolled and divided into three groups: AD (*n* = 27), MCI (*n* = 35), and NC (*n* = 40) (see Table 1). The mean age of AD group was the highest (67.70 ± 8.12 years), which was followed by the MCI group (65 ± 9.04 years). The median number of years of education was the lowest in the AD group (6 years). Compared to the NC group, the cognitive scores of the AD group were significant in every educational level subgroup, but were significant for the MCI group only in individuals with ≥6 years of education (*P* < 0.05). Twelve (44.4%) patients in the AD group had a CDR score of 2, and 15 (53.6%) had a score of 1.

**TABLE 1:** Participant characteristics.

Variables	Total (*n* = 102)	AD (*n* = 27)	MCI (*n* = 35)	NC (*n* = 40)	*p*-value
Sex: female**, *n* (%)	63 (61.8%)	19 (70.4%)	21 (60%)	18 (45.0%)	0.111
Age* (years), mean (SD)	63.98 (9.00)	67.70 (8.12)	65.00 (9.04)	60.58 (8.48)	**0.004^b^**
Height* (cm), mean (SD)	158.68 (7.14)	157.81 (7.16)	158.20 (7.19)	159.68 (7.15)	0.519
Weight* (kg), mean (SD)	57.70 (8.08)	58.15 (8.67)	58.46 (8.59)	56.73 (7.29)	0.620
Body mass index* (kg/m^2^), mean (SD)	22.89 (2.69)	23.26 (2.40)	23.37 (3.25)	22.22 (2.21)	0.127
Years of education** (years), median (IQR)	9 (6, 12)	6 (6, 9)	9 (6, 12)	9 (9, 12)	**0.010^b^**
MMSE scores**, median (IQR)	27 (21.75, 28)	15 (11, 20)	26 (24, 27)	29 (28, 30)	**<0.001^abc^**
≤5 years of education (*n* = 11)	23 (14, 24)	12.5 (9.5, 16.25)	23 (22, 24)	27 (27, 27)	**0.013^b^**
6∼12 years of education (*n* = 57)	26 (19.5, 28)	15.5 (11.75, 19.25)	26 (24.25, 27)	28 (28, 29)	**<0.001^abc^**
≥12 years of education (*n* = 34)	28 (27, 30)	20 (8, 22)	27 (27, 28)	30 (29, 30)	**<0.001^bc^**
MoCA scores**, median (IQR)	23 (15, 27)	11 (5, 15)	21 (17, 23)	27.5 (26, 29)	**<0.001^abc^**
≤5 years of education	15 (5, 16)	5 (4.25, 9.5)	15 (14.5, 15.5)	26 (26, 26)	**0.012^b^**
6∼12 years of education	21 (14.5, 26)	12.5 (7, 15)	20.5 (17.25, 23)	26 (26, 28)	**<0.001^abc^**
≥12 years of education	26.5 (23, 29)	14 (4, 19)	23.5 (22.5, 24.25)	28 (28, 29)	<**0.001^bc^**

*One way ANOVA. Bonferroni’s method was used for post hoc comparisons, and *P* < 0.05 indicated a significant difference between the two groups.

**Kruskal Wallis tests, and a two-by-two comparison was performed. An adjusted *P* < 0.05 indicated a significant difference between the two groups.

^*a*^Significant difference between AD and MCI.

^*b*^Significant difference between AD and NC.

^*c*^Significant difference between MCI and NC. AD, Alzheimer’s disease; MCI, mild cognitive impairment; NC, normal control; SD, standard deviation; IQR, interquartile range; MMSE, mini-mental state estimate; MoCA, Montreal cognitive assessment. Bold values indicate *P* < 0.05.

### 3.2 Descriptive analysis of gait parameters during ST and DT walking

The gait parameters were compared during ST and DT walking (see Table 2). Age and education were included as covariates. During ST gait, arm peak velocity differed significantly between the AD and NC groups, and between the MCI and NC groups (*P* < 0.05). The asymmetry of stride length and stride velocity differed significantly between AD and NC and between AD and MCI (*P* < 0.05). During DT walking, changes in gait were observed in the AD and MCI groups compared to the NC group, involving slow arm peak velocity, decreased arm motion, and high variability (*P* < 0.05). High asymmetry was exclusively observed between the AD and NC groups. However, *post hoc* comparisons indicated no significant difference between the AD and MCI groups.

**TABLE 2:** Descriptive data of gait parameters.

Variables	Single-task				Dual-task			
	AD (*n* = 27)	MCI (*n* = 35)	NC (*n* = 40)	*P*-value	AD (*n* = 27)	MCI (*n* = 35)	NC (*n* = 40)	*P*-value
Mean stride velocity (cm/s), mean (SD)	66.93 (22.08)^a^	83.56 (15.68)	85.45 (20.48)	**0.012**	52.41 (17.18)^b^	64.15 (18.05)	76.19 (21.02)	**0.001**
Stride length (cm), mean (SD)	81.61 (19.75)^a,b^	97.20 (17.11)	100.03 (16.98)	**0.006**	70.99 (17.54)^b^	83.01 (18.88)	92.78 (16.30)	**0.002**
Gait cycle time (s), mean (SD)	1.25 (0.13)	1.19 (0.10)	1.19 (0.11)	0.220	1.41 (0.22)^b^	1.36 (0.21)	1.24 (0.14)	**0.007**
Swing (%), mean (SD)	38.17 (2.62)	39.42 (1.99)	39.05 (2.46)	0.184	36.24 (3.59)	37.42 (2.25)	37.58 (2.69)	0.316
Double support (%), mean (SD)	24.54 (5.11)	22.29 (3.77)	22.97 (4.54)	0.211	28.54 (6.49)	26.16 (4.39)	25.18 (4.89)	**0.095**
Arm peak velocity (°/s), mean (SD)	134.89 (39.03)^b^	145.55 (47.38)^c^	197.27 (75.91)	**0.001**	125.87 (33.72)^b^	130.77 (41.24)^c^	193.29 (68.83)	**<0.001**
Arm range of motion (°), mean (SD)	44.67 (14.76)	38.83 (11.01)	43.60 (6.31)	0.070	37.75 (12.03)^b^	38.03 (11.33)^c^	44.17 (7.95)	**0.015**
Variability stride velocity (cm/s), mean (SD)	9.25 (3.67)	8.13 (3.04)	7.60 (1.72)	0.187	11.41 (4.10)^b^	11.05 (3.59)	9.22 (2.70)	**0.015**
Stride length (cm), mean (SD)	9.11 (4.40)	7.41 (3.11)	6.85 (1.70)	0.050	11.41 (4.33)^b^	10.69 (4.09)^c^	8.08 (2.69)	**0.001**
Gait cycle time (s), median (IQR)*	0.07 (0.05, 0.10)^a^	0.05 (0.03, 0.06)	0.04 (0.03, 0.07)	**0.047**	0.16 (0.10, 0.23)^b^	0.11 (0.07, 0.20)^c^	0.06 (0.04, 0.12)	**0.001**
Swing (%), median (IQR)*	2.67 (2.14, 3.52)	2.04 (1.53.2.94)	2.00 (1.46, 2.45)	0.078	2.67 (2.14, 3.52)^b^	2.04 (1.53.2.94)^c^	2.34 (1.72, 3.01)	**0.001**
Double support (%), median (IQR)*	3.06 (2.55, 4.00)	2.64 (1.96, 3.80)	2.48 (2.01, 3.35)	0.215	3.98 (3.09, 6.53)^b^	3.98 (3.11, 4.98)^c^	2.94 (1.89, 3.58)	**0.001**
Arm peak velocity (°/s), median (IQR)*	37.04 (21.46, 63.73)	31.34 (25.46, 40.62)	30.07 (20.53, 50.28)	0.262	36.95 (23.93, 59.52)	34.11 (25.65, 50.47)	36.97 (22.87, 58.37)	0.565
Arm range of motion (°), median (IQR)*	6.20 (4.20, 7.47)	4.70 (3.96, 5.54)	4.74 (3.56, 6.30)	0.118	6.34 (5.74, 7.75)	5.65 (4.71, 7.30)	5.21 (4.62, 7.81)	0.185
Asymmetry stride velocity (%), median (IQR)*	8.44 (6.12, 11.49)^a,b^	5.89 (4.42, 8.22)	5.76 (4.51, 7.68)	**0.009**	9.81 (7.12, 13.92)^b^	8.11 (5.97, 10.69)	6.55 (4.66, 8.17)	**0.004**
Stride length (%), median (IQR)*	7.96 (4.44, 9.94)^a,b^	4.24 (3.21, 6.09)	3.96 (2.52, 6.43)	**0.010**	7.19 (5.42, 8.68)^b^	5.68 (4.09, 7.85)	4.39 (3.56, 5.60)	**0.003**
Swing phase (%), median (IQR)*	6.73 (4.77, 10.54)	6.15 (4.22, 9.60)	6.35 (4.50, 8.70)	0.959	9.45 (5.89, 15.13)	8.58 (5.84, 12.38)	6.56 (4.48, 9.77)	**0.307**
Stance phase (%), median (IQR)*	4.15 (2.98, 6.73)	4.37 (2.85, 5.94)	4.07 (3.04, 5.37)	0.850	5.61 (3.54, 8.55)	5.56 (3.66, 7.37)	4.15 (2.98, 5.64)	0.458
Arm peak velocity (%), median (IQR)*	25.73 (18.73, 35.00)	21.90 (16.66, 28.01)	22.42 (16.92, 31.04)	0.426	27.58 (24.21, 35.66)	25.22 (21.00, 33.20)	24.72 (20.89, 33.16)	0.496
Arm range of motion (%), median (IQR)*	35.34 (33.66, 37.45)	34.89 (32.83, 40.96)	34.60 (30.84, 38.54)	0.283	36.79 (34.37, 38.09)	36.94 (34.92, 39.35)	36.20 (31.66, 39.11)	0.051

Analysis of covariance. Age and education were included as covariates. Bonferroni’s method was used for post hoc comparisons, and *P* < 0.05 indicated a significant difference between the two groups.

*Transformed by natural logarithm.

^*a*^Significant difference between AD and MCI.

^*b*^Significant difference between AD and NC.

^*c*^Significant difference between MCI and NC. AD, Alzheimer’s disease; MCI, mild cognitive impairment; NC, normal control; SD, standard deviation; IQR, interquartile range. Bold values indicate P < 0.05.

### 3.3 Extracting gait domains using FA

The mean value and asymmetry of the arm range of motion, were excluded from FA because of their lack of correlation with other parameters. The final FA included 18 gait parameters. Each extracted PC represented a specific gait domain that was named according to the gait parameter with the highest loading on the PC. The FA of the ST extracted five independent gait domains: rhythm asymmetry, rhythm, pace asymmetry, arm motion, and variability, explaining 77.16% of the total variance (see Table 3). The FA of DT extracted four independent domains: rhythm, variability, asymmetry and arm motion, explaining 72.03% of the total variance (see Table 4).

**TABLE 3:** Factor analysis of single-task gait.

Variables	Rhythm asymmetry	Rhythm	Pace asymmetry	Arm motion	Variability
Stride velocity		−0.600	−0.664		
Stride length			−0.704		
Gait cycle time		0.764			
Swing		−0.873			
Double support		0.878			
Arm peak velocity				0.619	
Stride velocity variability					0.917
Stride length variability					0.844
Gait cycle variability					
Swing variability	0.829				
Double support variability	0.707				
Arm peak velocity variability				0.852	
Arm range of motion variability				0.708	
Stride velocity asymmetry			0.858		
Stride length asymmetry			0.926		
Swing asymmetry	0.887				
Stance asymmetry	0.902				
Arm peak velocity asymmetry				0.706	

Varimax rotation. Only the absolute value of the loading greater than 0.5 is displayed.

**TABLE 4:** Factor analysis of single-task gait.

Variables	Rhythm	variability	Asymmetry	Arm motion
Stride velocity	−0.837			
Stride length	−0.682			
Gait cycle time	0.689			
Swing	−0.869			
Double support	0.887			
Arm peak velocity	−0.521			0.52
Stride velocity variability		0.927		
Stride length variability		0.868		
Gait cycle time variability	0.505			
Swing variability			0.54	
Double support variability	0.577			
Arm peak velocity variability				0.833
Arm RoM variability				0.719
Stride velocity asymmetry		0.576		
Stride length asymmetry		0.546		
Swing asymmetry			0.867	
Stance asymmetry			0.933	
Arm peak velocity asymmetry				0.652

Varimax rotation. Only the absolute value of the loading greater than 0.5 is displayed.

### 3.4 Association between gait domains and cognitive performance

In the linear regression models (see Supplementary Tables 2, 3), the gait domains were included as predictors of the MoCA score. Age and years of education were also included because of their significant differences between the groups. In ST gait, rhythm asymmetry, pace asymmetry, and variability were significantly correlated with MoCA scores in the entire sample (*P* < 0.05). In DT gait, rhythm and variability were significantly associated with MoCA scores in the entire sample (*P* < 0.05). No significant correlation was detected between arm motion and MoCA scores in both ST and DT gait (*P* > 0.05).

### 3.5 Utilizing gait domains to identify AD and MCI

**FIGURE 2:**
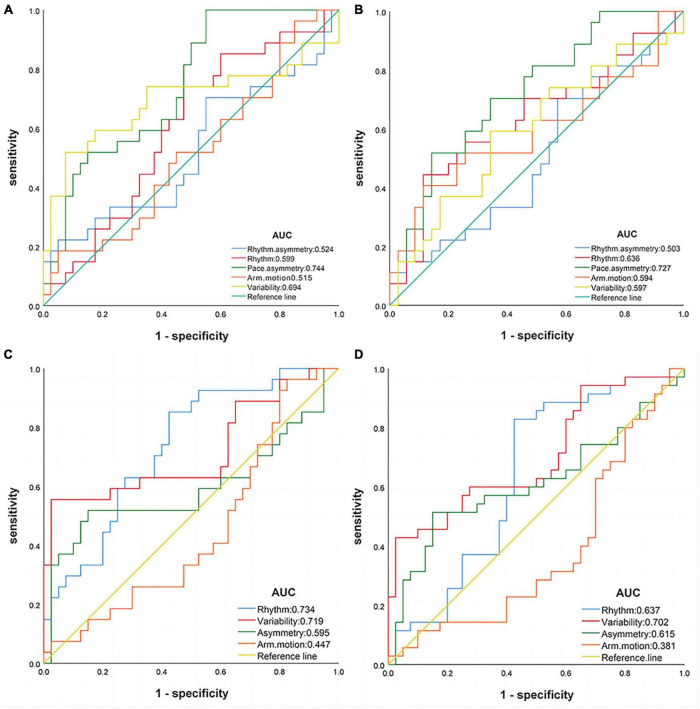
Receiver operating characteristic curves of gait domains. **(A)** Single-task: AD vs. NC; **(B)** single-task: AD vs. MCI; **(C)** dual-task: AD vs. NC; **(D)** dual-task: MCI vs. NC. AD, Alzheimer’s disease; MCI, mild cognition impairment; NC, normal control.

Receiver operating characteristic curves were used to evaluate the diagnostic efficacy of the gait domains (see Figure 2). The classification was deemed accurate based on the criteria of AUC ≥ 0.7 and *P* < 0.05. In the ST state, pace asymmetry could be used to distinguish between the AD and NC groups (AUC = 0.744, 95% CI: 0.628–0.861, *P* = 0.001, sensitivity: 100.0%, specificity: 45.0%); and between the AD and MCI groups (AUC = 0.727, 95% CI: 0.602–0.852, *P* = 0.002, sensitivity: 51.9%, specificity: 85.7%). In the DT state, rhythm (AUC = 0.734, 95% CI: 0.615–0.854, *P* = 0.001, sensitivity: 85.2%, specificity: 57.5%) and variability (AUC = 0.719, 95% CI: 0.583–0.854, *P* = 0.003, sensitivity: 55.6%, specificity: 97.5%) could discriminate AD from NC. Notably, only variability in DT was able to discriminate MCI from NC (AUC = 0.702, *P* = 0.003, 95% CI: 0.582–0.823, sensitivity: 42.9%, specificity: 97.5%). Arm motion did not exhibit effective discrimination in either task state. After adjusting for age and education, the AUC areas of pace asymmetry, variability, and rhythm were reduced (see Supplementary Figure 1).

## 4 Discussion

In this study, we discovered that during ST walking, gait asymmetry was negatively correlated with MoCA scores and could differentiate AD from the other two groups. However, we did not find a significant association between arm motion and MoCA scores in the multiple linear regression model. In addition, arm motion did not show good discrimination in either task.

### 4.1 Arm motion

To our knowledge, this study is the first to detect decreased arm peak velocity in patients with AD and MCI during both ST and DT walking. Decreased arm range of motion was also detected in the AD and MCI groups with the addition of cognitive task. Similar changes have been observed in the DT gait of patients with Parkinson’s disease (Baron et al., 2018). Arm-swing movements are important for coordinating walking stability (Meyns et al., 2013). Arm-swing amplitude typically has a positive correlation with walking speed (Kubo et al., 2004). In a study by Matuszewska and Syczewska (2023) the arm-swing amplitude was greater, more repetitive and symmetrical during fast walking than that during slow walking. This implies that changes in arm speed and amplitude may be driven by alterations in walking speed.

Arm movements can be influenced by cognitive load, possibly due to shared cortical networks involved in gait control and cognition (Krasovsky et al., 2014; Cohen and Verghese, 2019). However, our study found no significant association between arm motion and MoCA scores after considering other gait domains, age and education, suggesting that arm movements may passively respond to changes in other gait domains. For instance, the body performs preceding postural controls, such as adjusting the amplitude, rhythm and direction of the arm swing to prevent deterioration of balance and falls (Takakusaki, 2023). Several studies have highlighted the importance of arm swing in gait recovery (Bruijn et al., 2010; Krasovsky et al., 2014; Punt et al., 2015). Gait without arm swing is characterized by a higher perturbation resistance but similar local stability to gait with arm swing (Bruijn et al., 2010). This suggests arm swing may not be necessary for walking, but recovery movement of arm may contribute to overall gait stability. Our study did not intentionally set up obstacles to cause sudden gait disturbances and thus may have missed the key function of arm swing. A recent study suggested that right arm swing attenuation appears to be the norm in humans performing motor-cognitive tasks (Killeen et al., 2017). Distinct neural control mechanisms are likely utilized for the movements of the dominant and non-dominant arms (Sainburg and Kalakanis, 2000). Therefore, it is advisable to observe arm swing in overall gait performance rather than isolation, and pay more attention to lateralised effects in future studies.

### 4.2 Gait asymmetry

Gait asymmetry has previously been used to assess unilateral or asymmetric pathological changes such as hemiplegia, Parkinson’s disease, and dementia with Lewy bodies (Mc Ardle et al., 2019, 2020; Di Biase et al., 2022). However, our gait assessment detected increased asymmetry of stride velocity and stride length in the AD group during ST and DT walking, which is consistent with the findings of two previous studies (Maquet et al., 2010; Ghoraani et al., 2021). Ardle’s study detected swing time asymmetry but not stride length asymmetry in individuals with AD (Mc Ardle et al., 2020). The method for calculating asymmetry may be the main cause of the differences among studies. We used the relative deviation between the left and right feet rather than the absolute difference. Inconsistencies in the measurement equipment are also worth considering. The type, number, and placement of sensors may affect the accuracy of the motion data (Weizman et al., 2021).

During ST walking, we discovered that gait asymmetry was negatively correlated with MoCA scores and suggested that pace asymmetry, could be used to identify patients with AD. However, our study included mild and moderate dementia, and the applicability of gait assessment in severe dementia requires further investigation. Other possible confounding factors such as sex and comorbidities, should also be considered in future studies.

Compared to the AD group, the MCI group displayed fairly balanced gait symmetry during both ST and DT walking, which is inconsistent with previous studies (Ghoraani et al., 2021; Liu et al., 2021). Including age as a covariate significantly influenced the results. In Ghoraani et al. (2021) study, the healthy group had a younger age profile, but the analysis did not account for this potentially confounding factor. Liu et al. (2021) study indicated that stance phase asymmetry in MCI group decreased from performing the count backward task to engaging in more complex tasks, such as animal naming and subtracting three continuously. Gait asymmetry appears to become more noticeable as cognitive load increases. However, differences in education were not taken into account in these two studies, which may have impacted the consistency of cognitive load during DT gait. As for recognizing MCI, gait asymmetry performed worse than gait variability. Numerous studies have exhibited that DT gait variability in MCI patients is a highly promising gait marker for MCI diagnosis (Montero-Odasso et al., 2012; Lee and Park, 2018; Boripuntakul et al., 2022; Lindh-Rengifo et al., 2022).

### 4.3 Effects of cognitive tasks, age, and education on gait

The choice of cognitive task may affect the sensitivity of DT gait. The DT paradigm for cognitive tasks considers four main abilities: working memory, verbal fluency, attention, and visuospatial abilities (Ramírez and Gutiérrez, 2021). A meta-analysis found that the largest increase in DT cost occurred during continuous subtraction or verbal fluency tasks, with correspondingly greater motor-cognitive interference with step speed (Bishnoi and Hernandez, 2021). A higher cognitive load is beneficial to MCI recognition, since simple tasks may lead to ceiling effects (Bishnoi and Hernandez, 2021). Conversely, for the AD group, the successive minus seven task posted a challenge. Majority of patients with AD achieved less than three correct answers during walking in our study. These results suggest that a DT gait with a high cognitive load is critical for identifying MCI. However, for patients with AD, basic cognitive tasks like countdown may be more appropriate.

Age was a primary confounding factor in our study, resulting in an overestimation of the diagnostic efficacy of the gait domain. Aging significantly affects gait performance and increases the risk of falls (Li et al., 2018). With age, gait becomes increasingly dependent on executive functions, especially switching abilities (Li et al., 2018). Older individuals may recruit supplementary cortical and sub-cortical areas to compensate for degenerative changes in the brain during motor preparation and execution (Poirier et al., 2021). Thus, impaired cognition hinders the motor compensation through cognitive processes, may leading to poorer gait performance. A Chinese cohort study demonstrated the continued significance of the longitudinal reciprocal association between gait speed and cognition after adjusting for baseline age, gait speed, cognition, and potential con-founders (Li et al., 2023). In conclusion, the impact of age on gait should be considered cautiously, given that aging may lead to decreased cognition and motor functions (Collyer et al., 2022).

Education was also a potential influencing factor on DT gait performance. In the present study, the AD group was less educated than the NC group. In the same group, participants with higher educational levels exhibited better cognitive performance. Education is the core of cognitive reserves, which is associated with better cognitive performance and delayed onset of clinical symptoms of AD (Pettigrew et al., 2023). Education cultivates the knowledge, skills, and abilities necessary for continued participation in intellectually demanding activities, which means continuous new information processing and cognitive stimulation (Parisi et al., 2012). Therefore, for well-educated participants, the cognitive task may fail to offer appropriate cognitive load. Thus, we also suggest that the task design of DT gait takes educational attainment into account in future studies. In summary, cognitive assessment in older adults requires a multifactorial approach.

## 5 Limitations

This study has the following limitations. Referring to the previous literature, we selected only a few gait parameters for analysis. However, the MATRIX system 2.0 also generated numerous variables for the trunk and lower leg, which deserves further exploration in future studies. Moreover, the present study was a cross-sectional study, and was unable to determine the sequential relationship between gait changes and cognitive decline. Some potential confounding factors such as age, sex, education, comorbidities, and medication use, should also be carefully evaluated. The selection of cognitive tasks according to different cognitive reverses and disease subtypes will further improve the specificity and accuracy of DT gait testing. In future studies, we will check the applicability of gait in large samples from various sources.

## 6 Conclusion

In this study, we explored whether arm swing and gait asymmetry could be used to recognize AD and MCI. The results revealed that there is no reliable evidence that arm movements can be used to identify AD and MCI. Gait asymmetry can be used as a potential gait marker for the diagnosis of AD, but not for MCI. Our results may aid in the diagnosis of clinical AD and MCI and provide theoretical evidence for gait-based screening of cognitive impairment.

## Data availability statement

The original contributions presented in the study are included in the article/Supplementary material, further inquiries can be directed to the corresponding author.

## Ethics statement

The studies involving humans were approved by the Ethics Committee of the Hospital of Chengdu University of Traditional Chinese Medicine. The studies were conducted in accordance with the local legislation and institutional requirements. The participants provided their written informed consent to participate in this study.

## Author contributions

ZL: Data curation, Methodology, Software, Writing – original draft, Writing – review and editing. JZ: Data curation, Investigation, Validation, Writing – review and editing. JL: Formal analysis, Investigation, Software, Validation, Writing – original draft. MS: Methodology, Supervision, Writing – review and editing. PL: Formal analysis, Validation, Writing – review and editing. ZH: Investigation, Writing – review and editing. SL: Investigation, Writing – review and editing. DY: Conceptualization, Funding acquisition, Project administration, Resources, Supervision, Writing – review and editing. JG: Software, Writing – review and editing.
