# Supplementary material for: Using dual-task gait to recognize Alzheimer’s disease and mild cognitive impairment: a cross-sectional study
Source: Front Hum Neurosci. 2023 Dec 19;17:1284805. doi: 10.3389/fnhum.2023.1284805 (PMC10770261; doi:10.3389/fnhum.2023.1284805)
Supplement: Supplementary file 1 [file Data_Sheet_2.docx]

Supplementary table 1. Descriptions of gait parameters

| Gait parameters | | Definition | |
| --- | --- | --- | --- |
| Mean | Averaging the values generated for all gait cycles | | |
|  | Stride velocity（m/s） | | Stride length divided by current gait cycle time. |
|  | Stride length（cm） | | The sagittal distance between two heel contacts of the same foot. |
|  | Stride time (s) | | The time interval between two heel contacts of the same foot. |
|  | Stance phase (%) | | Time elapsed between the initial contact (heel contact) and the last contact (toe off) of the same foot. |
|  | Swing phase (%) | | Time elapsed between the last contact of the current footfall to the first contact of the next footfall on the same foot. |
|  | Double stance phase (%) | | Time elapsed during both feet contact the ground. |
|  | Arm peak velocity (°/s) | | The maximum angular velocity of the arm swing. |
|  | Arm range of motion (°) | | Maximum angle of arm swing range. |
| Variability | Standard Deviation of the basic parameters for each gait cycle. | | |
| Asymmetry | The asymmetries of the remaining gait parameters are generated directly by the software according to the following equation:  Asymmetry= $Average（\frac{\left\vert\mathrm{left} - \mathrm{right} \right\vert}{Maximum \left( left ,right \right)}）$ | | |

Supplementary table 2. The linear regression model of ST

|  | B | β | t | P | F | Adjusted R^2^ |
| --- | --- | --- | --- | --- | --- | --- |
| Rhythm.asymmetry | -1.424 | -0.188 | -2.385 | **0.019** | 9.955 | 0.383 |
| Rhythm | -1.015 | -0.134 | -1.657 | 0.101 |  |  |
| Pace.asymmetry | -1.618 | -0.214 | -2.668 | **0.009** |  |  |
| Arm.motion | -0.767 | -0.101 | -1.290 | 0.200 |  |  |
| Variability | -2.108 | -0.279 | -3.485 | **0.001** |  |  |
| Age(years) | -0.061 | -0.073 | -0.860 | 0.392 |  |  |
| Years of education | 0.736 | 0.372 | 4.510 | **＜0.001** |  |  |

Supplementary table 3. The linear regression model of DT

|  | B | β | t | P | F | Adjusted R^2^ |
| --- | --- | --- | --- | --- | --- | --- |
| Rhythm | -2.342 | -0.309 | -3.864 | **＜0.001** | 13.272 | 0.422 |
| Variability | -2.324 | -0.307 | -4.040 | **＜0.001** |  |  |
| Asymmetry | -1.206 | -0.159 | -2.055 | 0.043 |  |  |
| Arm.motion | 0.281 | 0.037 | .490 | 0.625 |  |  |
| Years of education | 0.766 | 0.388 | 4.882 | **＜0.001** |  |  |
| Age(years) | -0.056 | -0.066 | -.800 | 0.426 |  |  |

Supplementary figure 1. Adjusted receiver operating characteristic curves


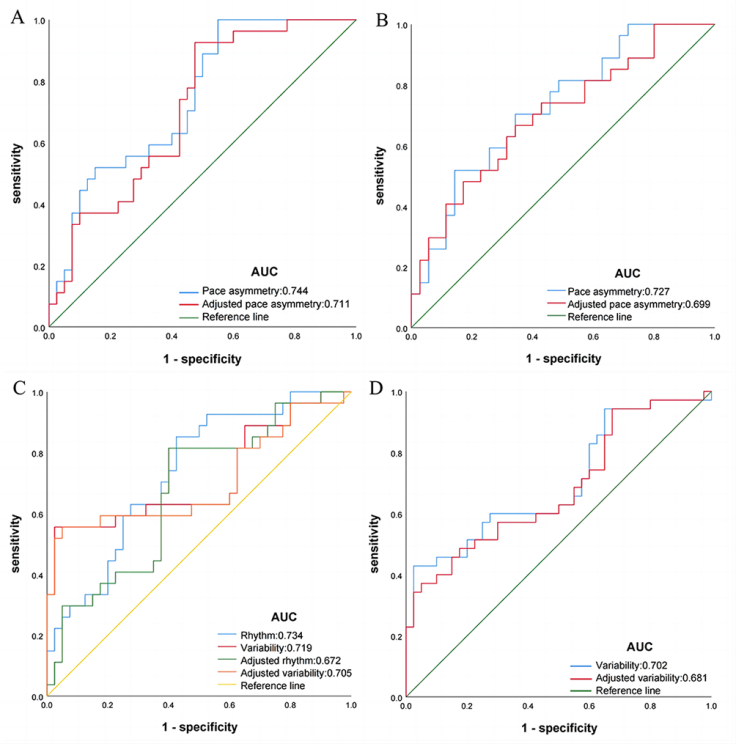


(A): single-task:AD vs NC; (B)single-task: AD vs MCI; (C) dual-task:AD vs NC; (D) dual-task: MCI vs NC

AD: Alzheimer’s disease; MCI: mild cognition impairment; NC: normal control
